# Young blood plasma reduces Alzheimer’s disease-like brain pathologies and ameliorates cognitive impairment in 3×Tg-AD mice

**DOI:** 10.1186/s13195-020-00639-w

**Published:** 2020-06-08

**Authors:** Ying Zhao, Ran Qian, Jin Zhang, Fei Liu, Khalid Iqbal, Chun-Ling Dai, Cheng-Xin Gong

**Affiliations:** 1 Department of Neurochemistry, Inge Grundke-Iqbal Research Floor, New York State Institute for Basic Research in Developmental Disabilities, 1050 Forest Hill Road, Staten Island, NY 10314 USA; 2 Department of Neurology, The Affiliated Huai’an Hospital of Xuzhou Medical University, Huai’an, 223001 Jiangsu China; 3 Department of Laboratory Diagnostics, School of Clinical Medicine, Hubei University of Science and Technology, Xianning, 437000 Hubei China; 4 Department of Rehabilitation, Guangzhou First People’s Hospital, School of Medicine, South China University of Technology, Guangzhou, 510180 Guangdong China

**Keywords:** Alzheimer’s disease, Cognitive function, Young blood plasma, Tau, Aβ, Neuroinflammation, Neurogenesis

## Abstract

**Background:**

Recent studies indicated that circulatory factors in blood plasma from young animals can reactivate neurogenesis, restore synaptic plasticity, and improve cognitive function in aged animals. Here, we investigated if young plasma could have a possible therapeutic effect for treatment of Alzheimer’s disease (AD)-like pathologies and cognitive impairment in triple-transgenic AD (3×Tg-AD) mice.

**Methods:**

We intravenously injected plasma from 2- to 3-month-old C57BL/6 J wild-type mice into 16–17-month-old 3×Tg-AD mice twice a week for 8 weeks. The behavioral tests including open field, novel object recognition, Morris water maze, and reversal Morris water maze were conducted after 4-week plasma injections. The effect of young plasma on tau and Aβ pathologies and on the levels of synaptic proteins and neuroinflammation were assessed by Western blots and immunohistochemical staining.

**Results:**

Young plasma treatment improved short-term memory in the novel object recognition test and enhanced the spatial learning and memory in Morris water maze test and reversal Morris water maze test. Biochemical studies revealed that young plasma treatment reduced both tau and Aβ pathologies, as well as neuroinflammation in the mouse brain. However, we did not detect any significant changes in levels of synaptic proteins or the dentate gyrus neurogenesis in the mouse brain after the treatment with young plasma.

**Conclusions:**

These data indicate that young blood plasma not only ameliorates tau and Aβ pathologies but also enhances the cognitive function in 3×Tg-AD mice. These findings suggest that transfusion with young blood plasma could be a potentially effective treatment for AD.

## Background

Alzheimer’s disease (AD) is the most common form of dementia and is characterized by progressive loss of memory and cognitive functions. Extracellular senile plaques consisting of amyloid-β (Aβ) peptides, intracellular neurofibrillary tangles (NFTs) composed of abnormally hyperphosphorylated tau protein, and neuronal/synaptic loss are the major histopathological hallmarks of AD [1]. Despite extensive research in the last 3–4 decades, it is still unknown what causes sporadic AD, and there is no effective treatment available to prevent or treat AD. Currently, it has been widely accepted that one or more of genetic risk, lifestyle, epigenetic and metabolic factors, and environmental factors affect the initiation and progression of AD. The contributions of these factors affecting the risk and development of AD may differ greatly among individuals.

Recent studies demonstrated that circulatory factors in the blood of aged mice have detrimental effects on hippocampal neurogenesis and cognitive function in young animals [2, 3]. Importantly, the exposure of aged animals to young blood via parabiosis or young plasma transfusion can rejuvenate the stem cell function in muscle, liver, spinal cord, and brain in aged rodents [2–6]. Especially, the circulatory factors in young blood can restore synaptic plasticity and reverse age-related cognitive deficits in mice [2–6]. More recently, treatment with human umbilical cord plasma was reported to revitalize the hippocampal function and improve cognition in aged mice [7].

Aging is accompanied by a series of cellular and functional impairments which may drive vulnerability to neurodegenerative disease. Given aging is the most important risk factor for AD and other neurodegenerative diseases in the elderly, remodeling the vasculature, reactivation of adult neurogenesis, and restoring of synaptic plasticity may delay the onset or even prevent the progression of AD and improve cognitive function and quality of life [8, 9]. However, the effects of circulatory factors in young blood on AD-related pathologies and cognitive function have not yet been reported.

Here, we injected young plasma to 16–17-month-old 3×Tg-AD mice twice a week for 8 weeks and found that young plasma treatment decreased AD-related pathologies and improved cognitive function in aged 3×Tg-AD mice. Our results suggest potential efficacy of young blood plasma for the treatment of AD and support clinical trials to investigate the potential benefits of circulatory factors in young plasma on AD and other neurodegenerative diseases.

## Methods

### Antibodies and reagents

Primary antibodies used in this study are listed in Table 1. Chemicals and other reagents were purchased from Sigma-Aldrich (St. Louis, MO, USA) unless otherwise noted.

**Table 1:** Primary antibodies used in this study

Antibody	Type	Specificity	Phosphorylation sites	Source/reference
R134d	Poly-	Tau		[10, 11]
pS199	Poly-	P-tau	Ser199	Invitrogen
AT8	Mono-	P-tau	Ser202/Thr205	Thermo Fisher Scientific.
pT212	Poly-	P-tau	Thr212	Invitrogen
pT231	Poly-	P-tau	Thr231	Invitrogen
PHF-1	Mono-	P-tau	Ser396/404	Dr. P. Davies [12]
Synapsin-1	Poly-	Synapsin-1		Santa Cruz Biotechnology
Synaptophysin	Mono-	Synaptophysin		Millipore
PSD95	Mono-	PSD95		Cell Signaling Technology
GFAP	Mono-	GFAP		Millipore
Iba1	Poly-	Iba1		Abcam
CREB	Poly -	CREB		Cell Signaling Technology
p-CREB	Poly-	p-CREB	Ser133	Cell Signaling Technology
Doublecortin	Poly-	Doublecortin		Abcam (ab18723)
β-Amyloid	Mono-	Aβ		Cell Signaling Technology
NeuN	Mono-	NeuN		Millipore
GAPDH	Poly-	GAPDH		Santa Cruz Biotechnology

### Mice

Wild-type C57BL/6 J and homozygous 3×Tg-AD mice were originally obtained from the Jackson Laboratory. The 3×Tg-AD mice harbored human APP_SWE_ and tau_P301L_ transgenes with knock-in PS1_M146V_ under the control of the mouse Thy1.2 promoter and were created by Dr. Frank M LaFerla [13]. Both homozygous male and female 3×Tg-AD mice with the mixed C7BL/6;129X1/SvJ;129S1/Sv genetic background were bred in our institutional animal colony. Mice were housed (4–5 animals per cage) in pathogen-free facilities with 12-h light/12-h dark cycles. The female 3×Tg-AD mice develop amyloid plaques starting at about 9 months of age and NFTs starting about 12 months of age, respectively, and the pathologies are predominantly restricted to the hippocampus, amygdala, and cerebral cortex [14, 15]. Animal studies were approved by the Institutional Animal Care and Use Committee (IACUC) of New York State Institute for Basic Research in Developmental Disabilities (Staten Island, NY, USA) and were according to US PHS NIH guidelines.

### Plasma collection

Mouse plasma was collected from young C57BL/6 J mice (2–3 months old) by intracardial bleeding at time of euthanasia. Plasma was prepared from blood collected with sodium citrate, followed by centrifugation at 1000×*g* for 10 min. Plasma collected from male and female mice was mixed at 1:1 (vol/vol). Saline as the vehicle control also contained the same amount of sodium citrate.

### Animal treatment

**Fig. 1:**
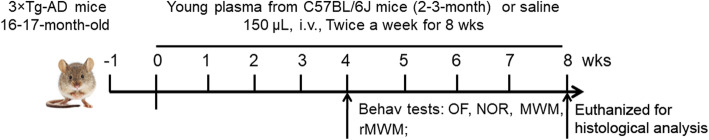
Study design. Female 3×Tg-AD mice were intravenously injected with 150 μl young plasma from 2- to 3-month-old C57BL/6 J mice (1:1 mixed from male and female) twice a week for 8 weeks. Behavioral tests including open field (OF), novel object recognition (NOR), Morris water maze (MWM), and reversal Morris water maze (rMWM) were carried out after the 9th plasma injection. All mice were sacrificed 24 h after the final injection; *n* = 10 for young plasma treatment, *n* = 11 for vehicle control (saline)

The female 16–17-month-old 3×Tg-AD mice were intravenously administrated through tail vein with 150 μl plasma twice a week for 8 weeks. The plasma was freshly prepared before injection. Behavioral tests were started on the second day after the 9th plasma injection (Fig. 1).

### Open field test

The open field test which is commonly used to assess simultaneously locomotion, exploration, and anxiety in rodents [16] was carried out as previously described [17]. Mice were transferred to the test room for habituation 1 h before starting the test. Each mouse was placed in the open field arena (made of opaque white plastic material, 50 cm × 50 cm, with walls 40 cm high) and allowed to explore the arena freely for 15 min. The distance traveled (meters) in open field arena and central area (10 cm × 10 cm), entries, and time spent in the central area were automatically recorded by a video-tracking system (Any maze version 4.5 software, Stoelting Co., Wood Dale, IL, USA).

### One-trial novel object recognition task

One-trial novel object recognition test was performed as described previously [10]. Briefly, the test consisted of a habituation phase, a sample phase, and a test phase. Following initial exposure, four additional 10-min daily habituation sessions were performed for mice to become familiar with the apparatus (50 × 50 × 40 cm) and the surrounding environment. On the fifth day, every mouse was first submitted to the sample phase of which two identical objects were placed in a symmetric position from the center of the arena. The mouse was allowed to freely explore the objects for 5 min. After a 20-min delay during which the mouse was returned to its home cage, the animal was reintroduced in the apparatus to perform the test phase. The mouse was then exposed to two objects for another 5 min: a familiar object (previously presented during the sample phase) and a novel object that had similar size as the familiar one but had different color and shape. Data was collected using a video-tracking system (ANY-Maze version 4.5 software; Stoelting Co., Wood Dale, IL, USA). Object discrimination index was calculated as follows: [(time spent exploring the novel object)/(time spent exploring both the familiar and the new objects) × 100%] during the test phase.

### Morris water maze task

Morris water maze (MWM) task was used to evaluate spatial learning and memory of the mice [10, 18, 19]. The test was performed in a circular white pool with a 180-cm diameter filled with non-toxic white dye tinted water and maintained at room temperature (20 ± 1 °C). The maze was designated of two virtual principal axes with each line bisecting the maze perpendicular to the other one to divide the maze into four equal quadrants. The end of each line demarcates four cardinal points: east, south, west, and north. A platform was positioned in the middle of one of the quadrants submerged 0.5 cm below water surface. Each mouse performed 4 trials for 4 consecutive days from semi-random start positions [20] to find the hidden platform. Each trial was terminated as soon as the mouse climbed onto the hidden platform. If a mouse failed to find the platform within 90 s, it was gently guided to the platform. At the end of each trial, the mouse was left on the platform for 10 s, then removed, dried, and returned to its home cage. A 90-s probe test without platform was performed 24 h after the last trial. Escape latency (s) in initial training and latency to 1st entrance into target (platform location area), target crossings, swim speed (cm/s), and time spent in target quadrant (s) in probe test were recorded through an automated tracking system (Smart video tracking system, version 2.0.14, Panlab; Harvard Apparatus).

### Reversal Morris water maze task

Reversal Morris water maze task was conduct 48 h after the probe test in the Morris water maze. Similar procedure was used as Morris water maze except the invisible platform was moved to the quadrant which was in an opposite location in the water maze. Probe test was conducted after 3 days of initial training.

### Immunofluorescence

Free-floating sagittal sections were washed three times for 15 min each in 10 mM PBS and incubated in PBS containing 0.3% Triton X-100 for 45 min. After blocking with a solution (5% normal goat serum, 0.1% Triton X-100, and 0.05% Tween 20 in PBS) for 2 h at room temperature, sections were incubated with primary antibodies mouse anti-doublecortin (Abcam, Ab18723, 1 μg/ml), AT8 (pSer202/Thr205, 0.1 μg/ml), or Aβ (D54D2, 1:1000) at 4 °C overnight. On second day, sections were washed three times for 15 min each with 10 mM PBS, followed by incubation with Alexa Fluor 488-conjugated goat anti-rabbit IgG or goat anti-mouse secondary antibody (1:1000; Molecular Probes, Eugene, OR, USA) in 10 mM PBS with 2% normal goat serum at room temperature for 2 h. Sections were subsequently washed, mounted, and coverslipped using ProLong Gold Antifade reagent. All images were acquired using a Nikon Eclipse Ti microscope (Nikon Instruments, Melville, NY, USA). Eight serial sections were analyzed in each mouse.

### Thioflavin-S staining

Thioflavin-S staining was performed as described previously [10]. Briefly, free floating brain sections were mounted on slides and dried at room temperature overnight. On the next day, slides with sections were rinsed in water for 6 min and then incubated in 0.25% KMnO_4_ for 4 min. After being washed in water for another 6 min, the sections were incubated in 1% K_2_S_2_O_5_ and 1% oxalic acid until the brown color completely faded. Then, sections were stained with 0.05% thioflavin-S in water for 8 min in dark. Finally, sections were washed in 80% ethanol for 2 min and in water for 3 min and coverslipped using FluoroGel mounting medium (Electron Microscopy Sciences, Hatfield, PA, USA). The maximum projection images were taken and thioflavin-S-positive plaques were quantified in subiculum and hippocampus using NIH Image J (v.1.46r). Eight serial sections were used from each mouse.

### Western blot analysis

Mouse brain tissue was homogenized in prechilled buffer containing 50 mM Tris-HCl (pH 7.4), 150 mM sodium chloride, 100 mM sodium fluoride, 1 mM sodium orthovanadate, 1 mM ethylene glycol-bis(β-aminoethyl ether)-N,N,N′,N′-tetraacetic acid (EGTA), 0.5 mM 4-(2-aminoethyl) benzenesulfonyl fluoride hydrochloride (AEBSF), 10 μg/ml aprotinin, 10 μg/ml leupeptin, and 10 μg/ml pepstatin. Each homogenate was boiled in 2× Laemmli sample buffer for 10 min, and the protein concentrations of the samples were measured by Pierce™ 660-nm protein assay (Thermo Scientific, Rockford, IL, USA). The protein patterns of samples were resolved by 8–12% SDS-PAGE dependent on the molecular weight of test proteins and electro-transferred onto Immobilon-P membranes (EMD Millipore, Billerica, MA, USA). The blots were then probed with primary antibodies (Table 1) and developed with the corresponding HRP-conjugated secondary antibody and enhanced chemiluminescence kit (Thermo Scientific, Rockford, IL, USA). Densitometric quantification of protein bands in Western blots was analyzed using Multi Gauge version 3.0 software (FUJIFILM North America, Valhalla, NY, USA).

### Statistical analysis

Data were analyzed using Prism version 5.0 software (GraphPad Software Inc., La Jolla, CA, USA). *T* test or two-way analysis of variance (ANOVA) (as appropriate) followed by a Bonferroni post hoc test was used. All data are presented as mean ± SEM. *p* < 0.05 was considered statistically significant.

## Results

### Treatment with young plasma does not affect body weight or anxiety

**Fig. 2:**
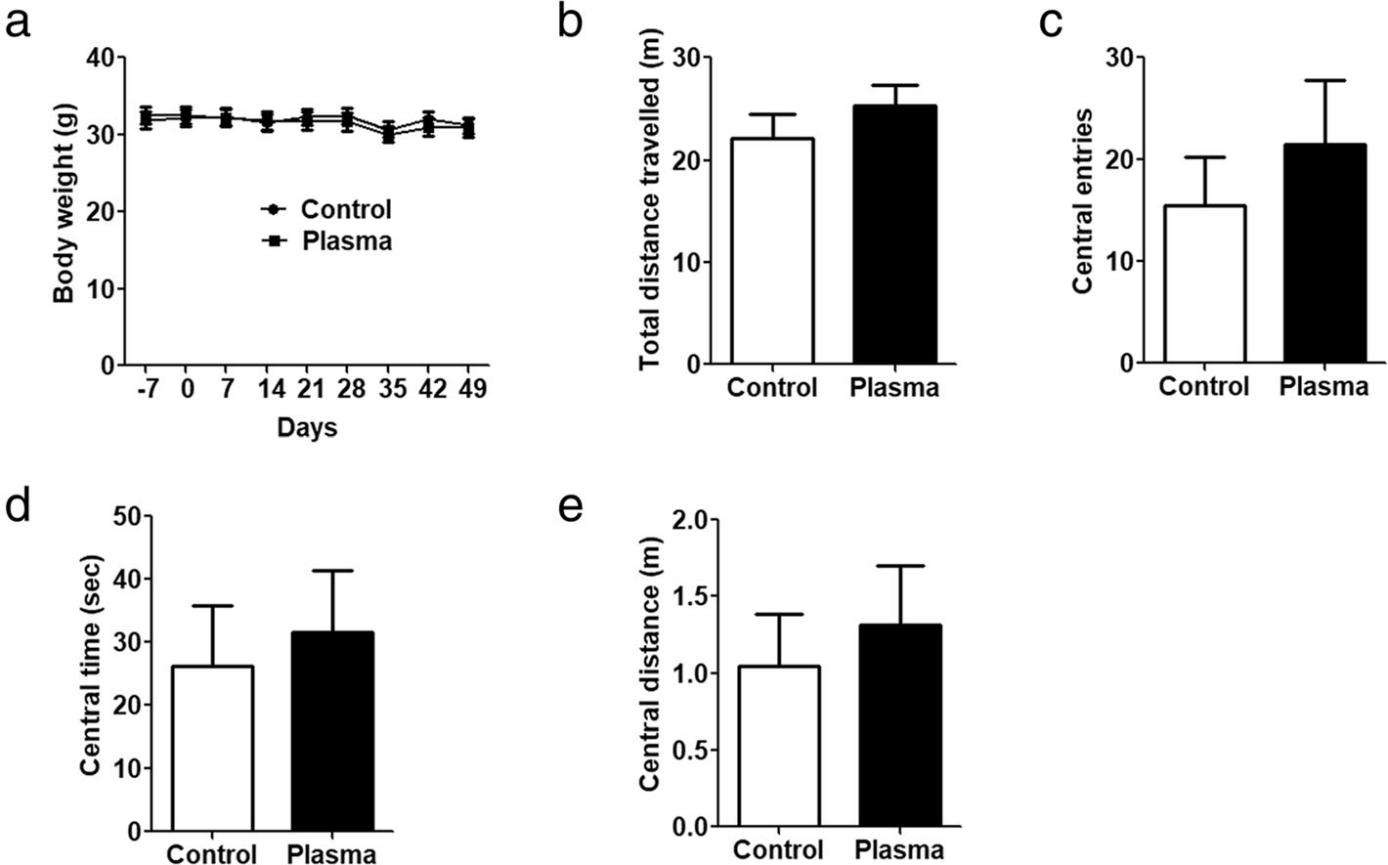
Body weight and open field test. **a** Body weight (two-way ANOVA, *F* = 0.44, *dfd* = 184, *p* = 0.8953). **b**–**e** Open field test. The total distance traveled (**b**), number of the central area entries (**c**), time in the central area (**d**), and distance traveled in central area (**e**) in an open field apparatus are presented. Unpaired *t* test was used for **b**–**e**, *n* = 10 for young plasma treatment, *n* = 11 for vehicle (saline) control

We studied the gross general health and the body weight every week for any possible effect of young plasma in 3×Tg-AD mice. We did not find any noticeable differences in the general condition or body weight between 3×Tg-AD mice treated with young plasma and with saline/vehicle control (Fig. 2a).

Because anxiety itself can affect tests for cognitive function [21], we first assessed the anxiety of 3×Tg-AD mice treated with young plasma or vehicle control. There were no significant differences in total distance traveled (Fig. 2b), number of central area entries (Fig. 2c), time in central area (Fig. 2d), and distance traveled in central (Fig. 2e) between the mice treated with young plasma and vehicle control. These results indicate that treatment with young plasma does not affect the general health or anxiety in 3×Tg-AD mice.

### Young plasma ameliorates cognitive impairment in aged 3×Tg-AD mice

**Fig. 3:**
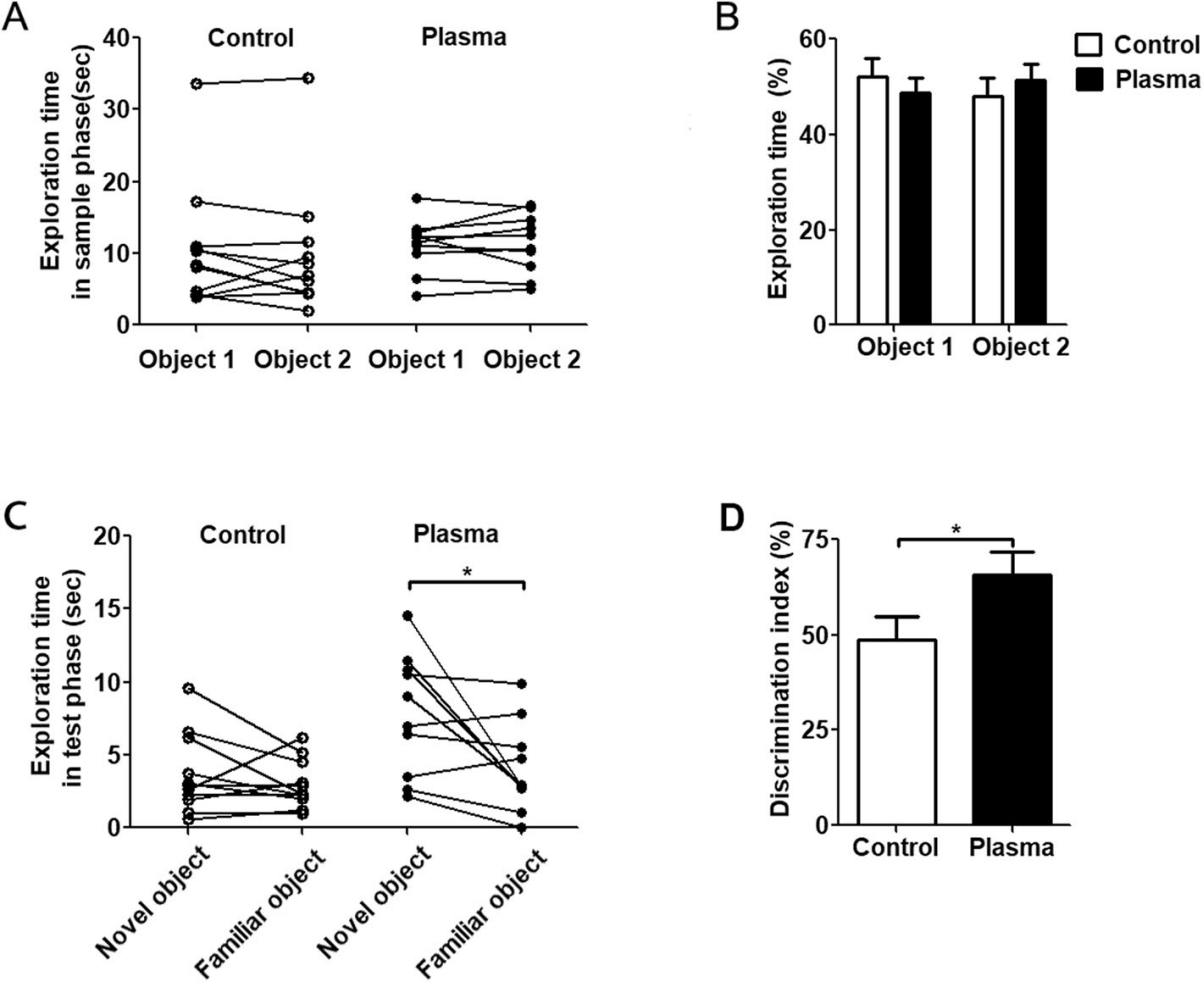
Young plasma improves short-term memory in one-trial novel object recognition test in 3×Tg-AD mice. **a** The time mice spent exploring two identical object 1 and object 2 in sample phase. **b** The percentage of time spent exploring two identical objects during sample phase. **c** The time of spent exploring novel object and familiar object during test phase. **d** Discrimination index (time spent exploring novel object/time spent exploring novel and familiar objects) × 100% in test phase. Paired *t* test was used for panels **a** and **c**, and unpaired *t* test for panels **b** and **d**, *p < 0.05. *N* = 10 for young plasma treatment, *n* = 11 for vehicle (saline) control

To explore whether young plasma can improve the cognitive dysfunction in aged 3×Tg-AD mice, we first conducted novel object recognition test to assess short-term memory. All mice treated with plasma or saline spent similar time on two identical objects in the sample phase (Fig. 3a and b), which indicated that the animal did not exhibit preference to the positions of objects. During the test phase, 3×Tg-AD mice treated with saline still spent similar time exploring the novel object and the familiar object (Fig. 3c and d), which indicated that short-term cognitive function of 3×Tg-AD mice was impaired. However, 3×Tg-AD mice treated with young plasma spent more time exploring the novel object than the familiar object during test phase (Fig. 3c and d). These results suggest that young plasma can ameliorate the short-term cognitive impairment in aged 3×Tg-AD mice.

**Fig. 4:**
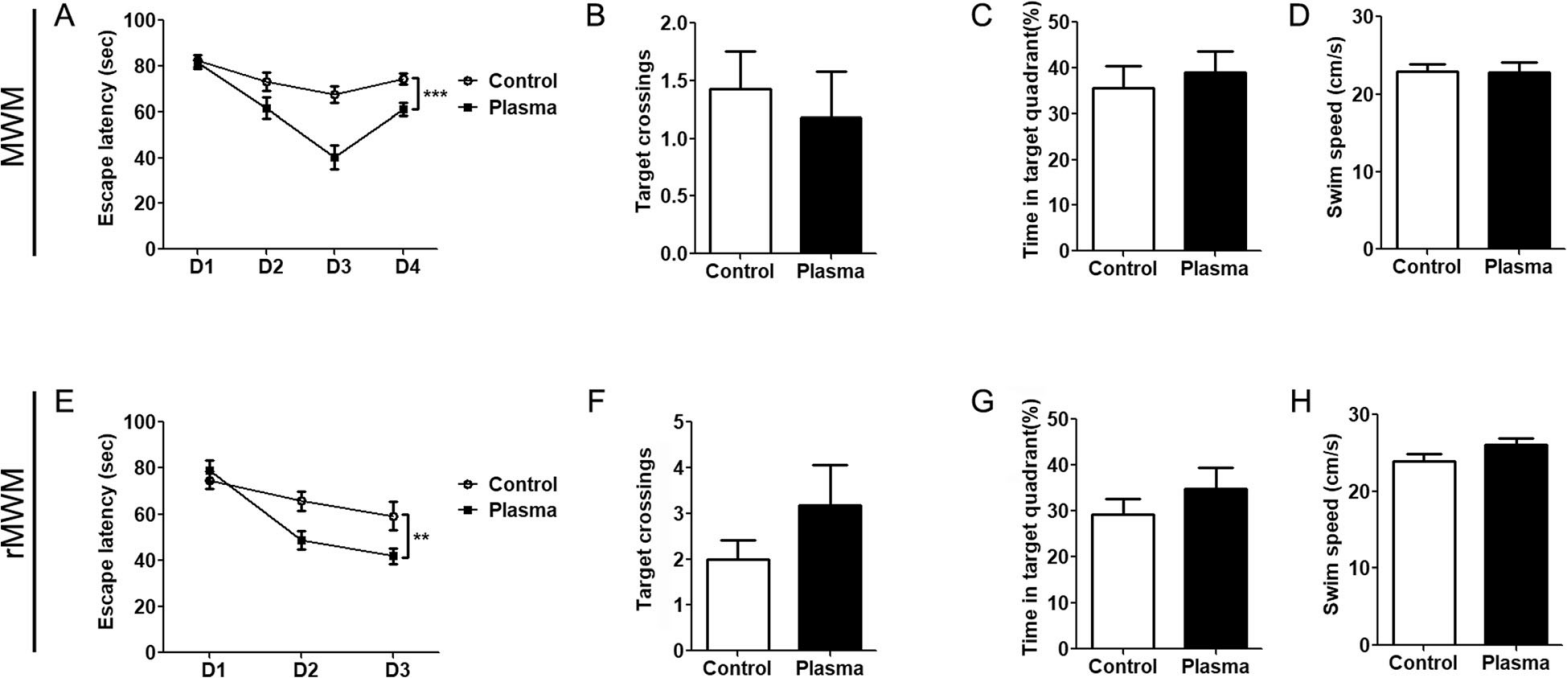
Young plasma improves spatial learning in 3×Tg-AD mice. **a**–**d**, Morris water maze test. The escape latency (**a**) to reach the hidden platform during acquisition phase for 4 days (two-way ANOVA, *F* = 9.77, *dfd =* 72*, p =* 0.0026), and the number of target area crossings (**b**), percentage of time in the target quadrant (**c**), and the average swim speed (**d**) of mice during the probe test. **e**–**h** Reversal Morris water maze test. The escape latency (**e**) to reach the hidden platform during acquisition phase for 3 days (two-way ANOVA, *F* = 17.17, *dfd =* 76*, p <* 0.0001), and the number of target area crossings (**f**), percentage of time in the target quadrant (**g**), and the average swim speed (**h**) during the probe test. Unpaired *t* test was used for **b**–**d** and **f**–**h**. *N* = 10 for young plasma treatment, *n* = 11 for vehicle (saline) control

We further investigated the effect of young plasma on the long-term spatial learning and memory by Morris water maze and reversal Morris water maze tests. During four consecutive days of the acquisition phase, 3×Tg-AD mice treated with vehicle did not show any decrease in time in reaching the platform (Fig. 4a), indicating a severe impairment in spatial learning. In contrast, the mice treated with young plasma showed a clear learning, as demonstrated by the decrease in the escape latency to reach the platform from day 1 to day 3 of the acquisition phase (Fig. 4a). Although the escape latency of the young plasma-treated mice was bounced back on day 4 of the acquisition phase for some unknown reason, it was still significantly shorter than that of the control mice. These data suggest that the young plasma ameliorates impairment of spatial learning in aged 3×Tg-AD mice. During the probe test, the 3×Tg-AD mice treated with young plasma or vehicle control did not show any significant differences in the number of crossings in the platform location (Fig. 4b) or the time spent in the target quadrant (Fig. 4c). The swim speed was not different between two groups of mice. Similarly, reversal Morris water maze test showed significant beneficial effect in young plasma-treated 3×Tg-AD mice in escape latency but not in probe test (Fig. 4e-h). Altogether, these results suggest that young plasma can enhance the ability of spatial learning and short-term memory but not the long-term spatial memory.

### Young plasma decreases tau pathology in 3×Tg-AD mice

**Fig. 5:**
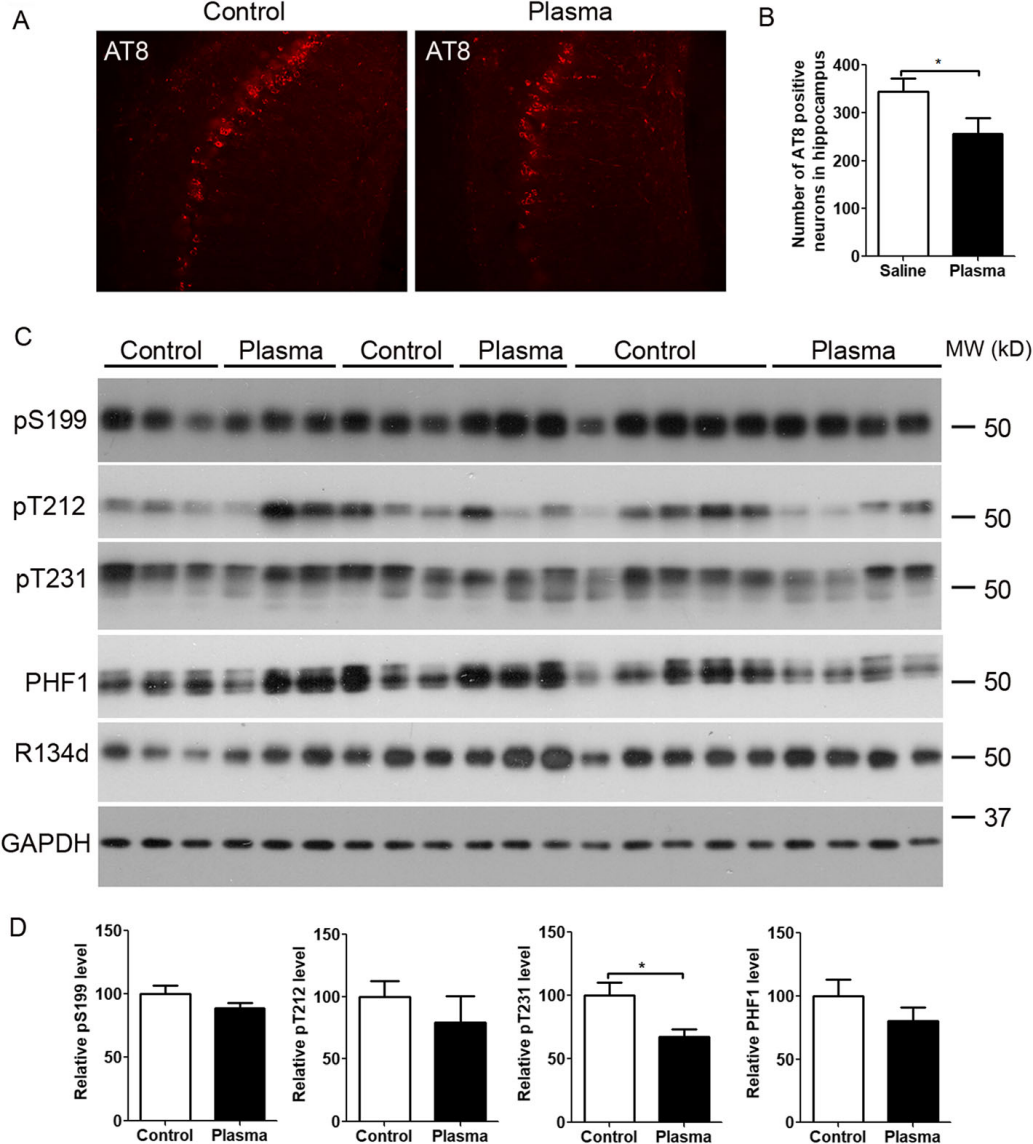
Yong plasma decreases tau pathology in 3×Tg-AD mice. **a**, **b** Immunostaining with AT8 antibody. **a** Representative images with AT8-positive staining. **b** AT8-postive neurons in CA1 were counted from 8 sections (from every 6th serial brain sections) of each mouse. *N* = 11 for saline control. *N* = 10 for young plasma treatment. **c**, **d** Representative Western blots of the hippocampus developed with R134d against total tau, and several phosphorylation-dependent and site-specific tau antibodies. Densitometric quantification of blots after normalization with R134d is shown. Unpaired *t* test was used for **b** and **d**, *p < 0.05. *N* = 10 for young plasma, and *n* = 11 for vehicle (saline) control

To investigate whether the treatment of 3×Tg-AD mice with young plasma for 2 months can influence tau pathology, we immunostained the mouse brain sections with AT8 antibody to tau pSer 202/pThr 205 and counted the number of neurons with positive AT8 staining in CA1 of the hippocampus. We found that young plasma treatment can reduce the number of neurons with AT8-positive staining (Fig. 5a and b). We also detected the levels of total tau with R134d antibody and phosphorylation of tau with several site-specific tau antibodies by Western blots. Young plasma treatment decreased the level of tau phosphorylation at threonine 231 and showed a trend to reduce tau phosphorylation at serine 396 and 404 with PHF1 antibody (Fig. 5c and d). These data suggest that young plasma can decrease tau pathology which may contribute to the beneficial effect on cognitive improvement.

### Young plasma decreases amyloid beta plaques in 3×Tg-AD mice

**Fig. 6:**
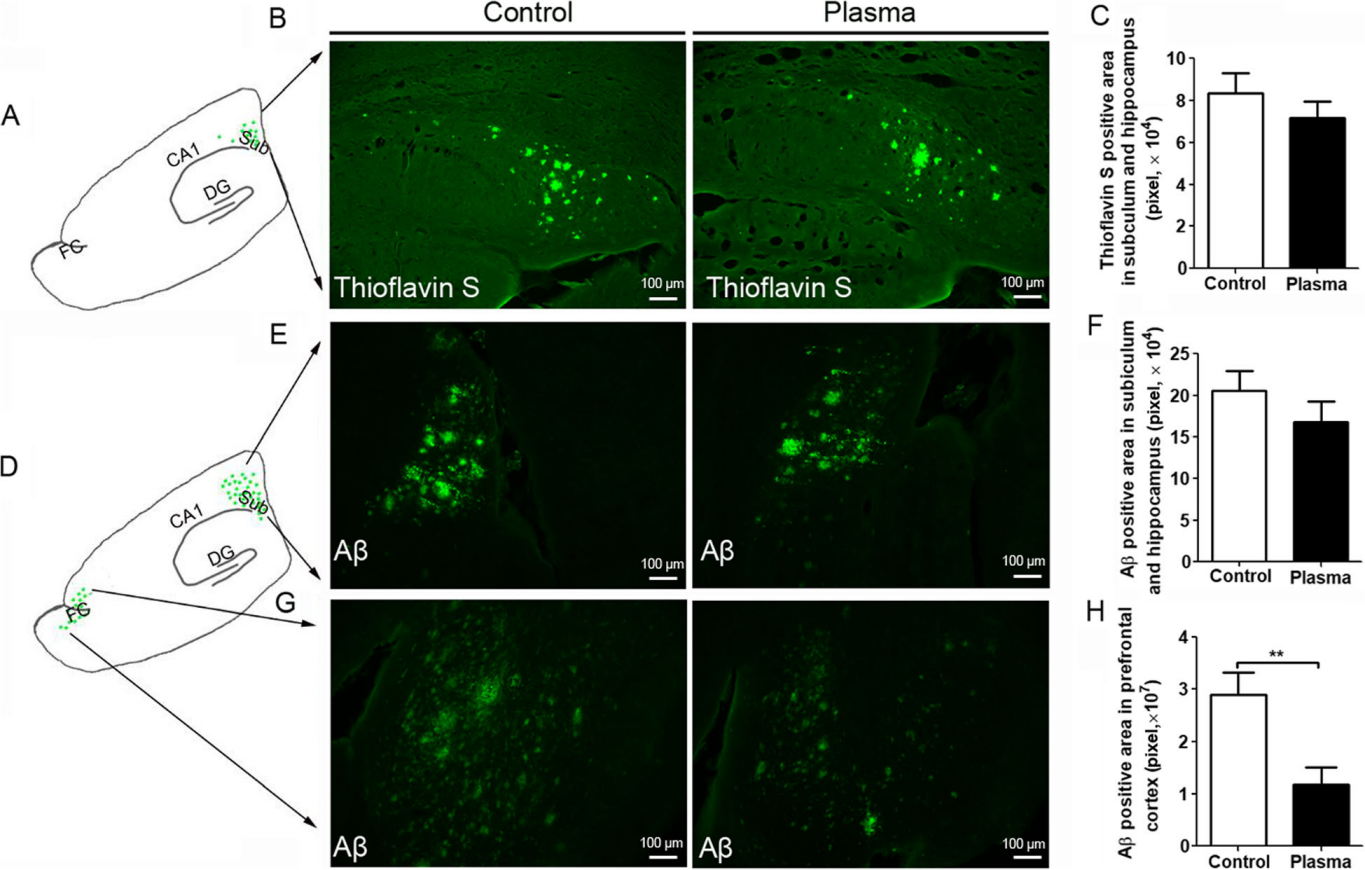
Young plasma decreases Aβ pathology in 3×Tg-AD mice. **a**–**c** Thioflavin-S staining in the subiculum and the hippocampal CA1 region of the mouse brain. **a** Schematic diagram for the thioflavin-S staining in the subiculum and CA1 region. **b** Representative images of the thioflavin-S staining. **c** Quantification of the thioflavin-S-positive amyloid plaque area. **d**–**h** Aβ staining in the subiculum and frontal cortex. **d** Schematic diagram for the Aβ staining. **e** Representative images of Aβ staining in the subiculum and CA1 region. **f** Quantification of the Aβ-positive amyloid plaque area in the subiculum and CA1 region. **g**, Representative images of the Aβ staining in the frontal cortex. **f** Quantification of the Aβ-positive amyloid plaque area in the frontal cortex. Amyloid plaque area was defined with a threshold using Otsu’s arithmetic with black and white in ImageJ. The actual amyloid plaque area was calculated by percent plaque area occupied × the whole image area containing the amyloid plaque, which avoided the bias from selecting the regions. The average of the plaque areas from 8 sections (every 6th serial brain sections) of each mouse was used for analysis. Unpaired *t* test was used for **c**, **f**, and **h**, **p < 0.01. *N* = 11 for vehicle (saline) control. *N* = 10 for young plasma treatment

The 3×Tg-AD mice start developing amyloid beta plaques as early as at 6-month-old of age. We employed thioflavin-S staining and immunofluorescence with Aβ antibody to investigate whether treatment with young plasma can reduce Aβ burden and/or prevent Aβ deposition. Thioflavin-S stains mature amyloid fibrils with a β-sheet conformation [22]. Thioflavin-S-positive Aβ plaques were mainly limited in the subiculum and CA1 area in 18–19-month-old 3×Tg-AD mice. The mice treated with young plasma showed a trend to reduce Aβ plaques area in the subiculum and the adjacent CA1 though it did not reach a significant difference (Fig. 6a-c). As expected, Aβ antibody staining revealed more amyloid plaques than thioflavin-S staining in the subiculum and CA1 area (Fig. 6b, c, e, and f). Similarly, young plasma treatment also exhibited a trend to reduce the plaque area in the subiculum and CA1 region (Fig. 6e and f). Interestingly, we did not find any thioflavin-S-positive staining except in the subiculum and in the CA1 region adjacent to the subiculum. However, we found Aβ-positive staining with Aβ antibody in the frontal cortex, and treatment with young plasma significantly reduced the amyloid plaque area in this brain region (Fig. 6g and h). These data indicate that young plasma may block the formation of Aβ plaques and/or promote their clearance.

### Young plasma inhibits brain inflammation in 3×Tg-AD mice

**Fig. 7:**
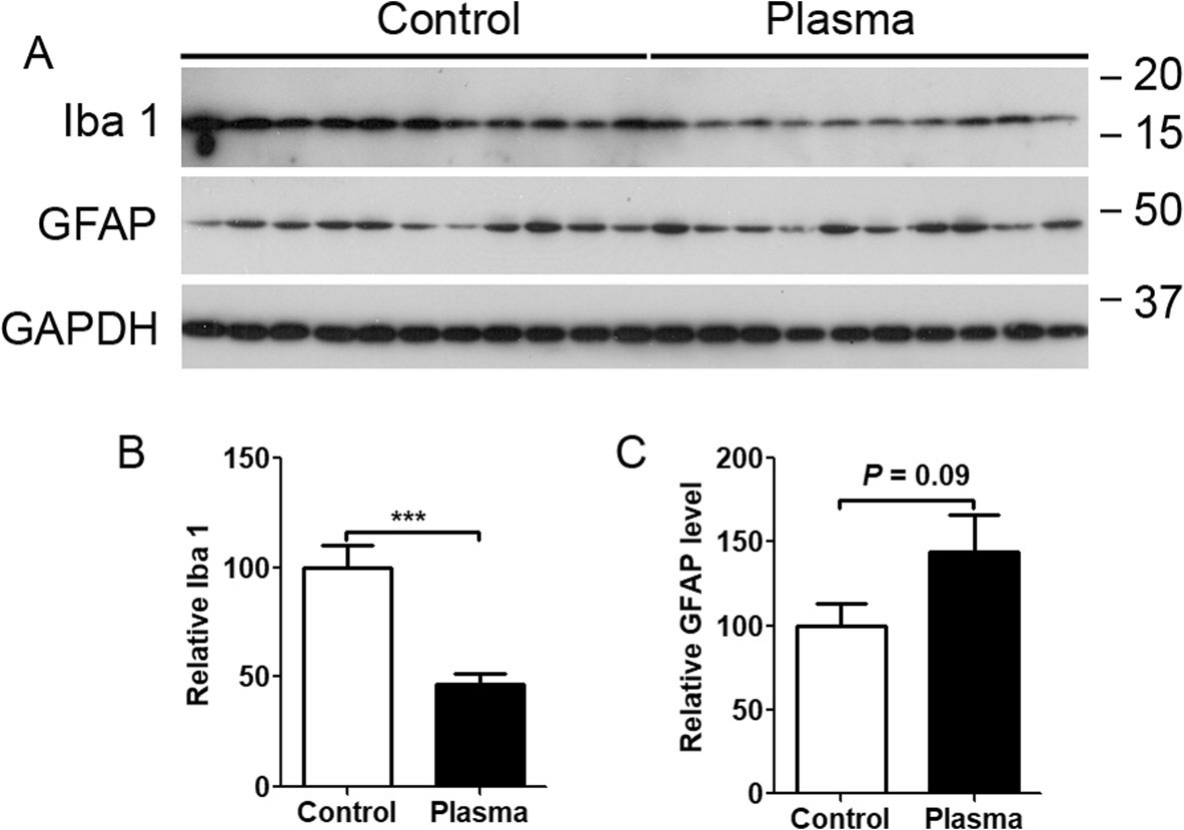
Young plasma decreases neuroinflammation in 3×Tg-AD mice. **a** Western blots developed with Iba1 and GFAP. **b** Densitometric quantification of blots after normalization with GAPDH is shown. Unpaired *t* test was used for **b** and **c**, ***p < 0.001. *N* = 10 for young plasma treatment, *n* = 11 for vehicle (saline) control

The 3×Tg-AD mouse brain exhibits neuroinflammation as early as in 3-month-old animals [23, 24]. Systemic factors can decrease the level of brain inflammation in APPswe/PS1dE9 mice [25]. We analyzed the levels of Iba 1, a microglia marker, and GFAP, an astrocyte marker, to assess whether young plasma can reduce neuroinflammation. We found that treatment with young plasma significantly decreased Iba 1 level in the hippocampus, suggesting that young plasma can inhibit neuroinflammation. Interestingly, treatment with young plasma showed a trend to increase the GFAP level (Fig. 7).

### Effect of young plasma on synapse-related proteins and on neurogenesis in 3×Tg-AD mice

Previous studies indicated that treatment of aged mice with young plasma can increase the level of synapse-related proteins [5, 26]. Here, we analyzed the levels of synapsin 1, synaptophysin, PSD95, cAMP response element-binding protein (CREB) and phosphorylated CREB (p-CREB), and neuronal marker NeuN by Western blots. However, we did not find any significant differences in the levels of these synapse-related or neuronal proteins in the hippocampal homogenates between the mice treated with young plasma and with vehicle control animals (supplementary Fig. 1). Immunohistochemical studies of the brain tissue sections did not show any significant differences in these proteins between the two groups either (data not shown). Additionally, we did not observe any newborn neurons with doublecortin staining in 3×Tg-AD mice treated with young plasma or vehicle control (data not shown). To exclude any possible antibody and method issues, which could otherwise lead to false negative, we stained the brain sections from C57BL/6 J mice, B6129SF2/J mice (having the same genetic background as 3×Tg-AD mice), and untreated 12-month-old 3×Tg-AD mice together with the 3×Tg-AD mice treated with young plasma or vehicle control. We found doublecortin positive neurons in the brain sections of the wild-type mice and in 12-month-old 3×Tg-AD mice, and no doublecortin-positive cells in the 18–19-month-old 3×Tg-AD mice treated with young plasma or saline.

## Discussion

Recent studies using parabiosis, young plasma transfusions, and systemic administration of identified blood factors indicate that circulatory factors in young blood can ameliorate deficits in adult neurogenesis, synaptic plasticity, and cognitive function in aged mice [2, 3, 5, 8, 27–29]. These age-related characteristic changes are also linked to AD and other neurodegenerative diseases and contribute to the impairment of brain function in AD. Currently, whether the circulatory factors in young blood can also rejuvenate the brain function in AD mouse model is unknown. A recent pilot human study, which assessed the safety, tolerability, and feasibility of infusions of young plasma in patients with AD, demonstrated that plasma administration in human is well tolerated and does not cause any serious adverse effects, but whether the treatment can benefit AD patients was inconclusive due to the small sample size and short duration of the study [30]. In the present study, we found that treatment with young plasma can ameliorate AD-related pathologies and improve cognitive function in aged 3×Tg-AD mice. These findings suggest that circulatory factors in young blood can reverse AD-like pathologies and cognitive impairment in aged 3×Tg-AD mice.

There are very few studies on the effect of circulatory factors in young blood on neurogenesis and cognitive function in AD mouse models. To date, only one study investigated the effect of young plasma on AD-related pathologies and cognition in APP mice that express mutant human APP (isoform 751) bearing both the Swedish (K670N/M671L) and the London (V717I) mutations under the control of the murine thy1 promoter [26]. Using parabiosis between 16- and 20-month-old APP mice and 2–3-month-old WT mice for 5 weeks, or treatment with young plasma from 2- to 3-month-old female WT mice to 10–12-month-old female APP mice twice a week for 4 weeks, circulatory factors in young blood were found to restore the level of synaptophysin and improve the cognitive function as assessed by Y-maze in these APP mice. However, young plasma did not have any effect on Aβ burden or neuroinflammation [26]. A recent study reported that parabiosis of APPswe/PS1dE9 mice with age-matched WT mice starting at the age of 3 months for 6 months (prevention study) or starting at 9-month-old for 3 months (treatment study) both significantly decreased Aβ burden in the mouse brains [25]. These data suggest that factors in the healthy circulation system can help prevent or reduce Aβ deposition in the transgenic mouse brains. Peripheral tissues and organs, especially the liver, kidney, gastrointestinal tract, and skin, may be involved in the clearance of Aβ, rather than through modulation of brain Aβ production, degradation, and receptor-mediated transport across the blood-brain barrier [25, 31]. Given that circulatory factors in young blood can rejuvenate the function of peripheral tissues such as liver and muscle [27, 32], which may contribute to the clearance of Aβ and thereby reduce its deposition in the brain, further studies are needed to explore the underlying mechanisms. In the present study, we found a significant lower level of Aβ deposition assessed by Aβ immunostaining in 3×Tg-AD mice treated with young plasma, but not by thioflavin-S staining, which assesses mature Aβ plaques in the mouse brain. It is noticed that we observed the thioflavin-S-positive amyloid plaques only in the subiculum and, to a less extent, in the adjacent CA1 region of the hippocampus in the 18–19-month-old 3×Tg-AD mice, whereas Nicholson et al. reported a wide distribution of amyloid plaques stained with thioflavin-S, including in the ventral/dorsal hippocampus, amygdala, and lateral septum/basal forebrain in these mice [33]. When we used similar staining condition as they used, we found wide-spread non-specific autofluorescence in the old mouse brains. We thus used 80-fold lower thioflavin concentration and also pretreated tissue sections with KMnO_4_ to reduce background autofluorescence before the thioflavin-S staining. We believe that the different distributions of thioflavin-S-positive amyloid plaques between the present study and the abovementioned study are probably due to different staining conditions used. Significant different amyloid and tau pathologies in the brains of 3×Tg-AD mice have also been reported by different laboratories previously.

Previous studies in aged animals reported that circulatory factors from young blood can reactivate the adult neurogenesis; restore synaptic plasticity by increasing levels of synaptic proteins such as synapsin 1, synaptophysin, and phosphorylation of CREB; and improve cognitive function. In the present study, we did not detect any beneficial effect of young plasma on synaptic proteins. One of the possible reasons is that the change in levels of synaptic proteins was undetectable by Western blots. Similarly, we did not find any increase in neurogenesis by doublecortin staining in the plasma-treated 3×Tg-AD mice. The normal aged mice still have the ability of neurogenesis under the stimulators from circulatory factors in young blood, although this capacity declines with aging. However, apparently, 3×Tg-AD mice have dramatically lost the ability of the dentate gyrus neurogenesis with aging as compared with WT mice. A previous study reported an almost completely impaired capacity for neurogenesis even in 12-month-old 3×Tg-AD mice [34]. We also found lots of doublecortin-positive cells in 12-month-old WT mice, fewer cells in 12-month-old 3×Tg-AD mice, and none doublecortin-positive cells in 18–19-month-old 3×Tg-AD mice employed in current study. The functionality of neurogenic niche may be impaired irreversibly in 3×Tg-AD mice at this age. In addition, short-term treatment such as 8 weeks might not be sufficient to promote the neurogenesis. These results suggest that long-term young plasma treatment may be required to rejuvenate the brain function in AD patients.

Parabiosis of APPswe/PS1dE9 mice with WT mice was found to result in the downregulation of inflammation by decreased levels of GFAP and CD45; reduction of proinflammatory cytokines including TNFα, IL-1, and IL-6; and the decrease of tau phosphorylation at Ser199 and Ser396 in AD mice [25]. In the present study, we found that young plasma can ameliorate the neuroinflammation and tau pathologies in aged 3×Tg-AD mice. Attenuation of inflammation and reduction of Aβ deposition in the mouse brain could have contributed to the reduction of tau phosphorylation in the brain.

Most importantly, in the present study, we found an apparent improvement in cognitive function after treatment with young plasma in 3×Tg-AD mice. The underlying molecular mechanism remains to be elucidated. The 3×Tg-AD mice are known to show neuroinflammation as early as at the age of 3 months [23, 35], which is prior to the onset of Aβ and tau pathologies and suggests that chronic inflammation may initiate and/or accelerate the progression of AD-like pathologies. Inhibition of inflammation leads to the reduction of Aβ and tau pathologies and improvement of cognitive function in 3×Tg-AD mice [36–38]. The present study demonstrates that circulatory factors in young blood can downregulate inflammation, reduce Aβ deposition, and decrease tau phosphorylation, which could have contributed to the reversal of cognitive impairment in aged 3×Tg-AD mice; 3×Tg-AD mice are known to become cognitively impaired as early as the age of 3 months [39].

A few circulatory factors in the young plasma were recently identified to regulate brain neurogenesis and cognitive function. C-C motif chemokine 11 (CCL11)/Eotaxin-1 was found to be an age-related systemic factor associated with decreased neurogenesis in healthy aged mice, and systemic administration of CCL11 to young adult mice was found to decrease adult neurogenesis and impair learning and memory [2]. Growth differentiation factor 11 (GDF11) is a transforming growth factor β protein that regulates aspects of central nervous system (CNS) formation and health throughout the lifespan [40]. Administration of recombinant GDF11 to aged mice can restore subventricular zone (SVZ) vascularization, enhance neurogenesis [3], and reverse age-related dysfunction in skeletal muscle [27] and cardiac hypertrophy [28]. Systemic administration of tissue inhibitor of metalloproteinases 2 (TIMP2), a blood-borne factor enriched in human cord plasma, young mouse plasma, and young mouse hippocampi, increases synaptic plasticity and hippocampal-dependent cognition in aged mice [7]. Additionally, growth hormone–releasing hormone (GHRH) has potent effects on brain function and likely plays a role in the pathogenesis of AD, and its level decreases with advancing age. GHRH administration modulates inhibitory neurotransmitter and brain metabolite levels and has favorable effects on cognition in both adults with MCI and healthy older adults [41, 42]. We also found that chromic oral treatment with a ciliary neurotrophic factor (CNTF) derived peptidergic compound (P021) to 3×Tg-AD mice can reduce Aβ production and rescue deficits in cognition, neurogenesis, and synaptic plasticity in 3×Tg-AD mice [43]. Although these identified factors exhibited important role in the regulation of brain neurogenesis and cognitive function as a single factor in different studies, it is likely that more plasma factors, including those not identified yet, work together to rejuvenate the brain function during aging.

Limitations of the present study include the absence of an additional control group treated with old plasma and the somewhat different genetic background of the young mice from which the plasma were collected. Although the present study is the first to use an AD mouse model to test this concept, previous studies using the same mouse strain for both donor and recipient mice have demonstrated that young plasma can indeed induce similar beneficial effects on old mice [2, 3, 5, 6]. On the other hand, circulatory factors in old plasma can impair cognitive function in young animals [2, 3]. We believe it is the circulatory factors in young plasma that contribute to the beneficial effects observed in the present study, although we could not exclude the function of infusion general proteins compared with the saline-treated group. Further parabiosis studies including isochronic parabiosis between young C57BL/6 J mice, isochronic parabiosis between aged 3×Tg-AD mice, and heterochronic parabiosis between young C57BL/6 J mice and aged 3×Tg-AD mice will help identify the role of the circulatory factors in young plasma in the beneficial effects in AD mouse model of 3×Tg-AD mice.

Consistent with other preclinical studies, we did not observe apparent adverse effects. There was no significant change of body weight in 3×Tg-AD mice treated with young plasma. Importantly, one pilot human study demonstrated that plasma transfusion (250 ml, once weekly for 4 weeks) was well tolerated and there were no serious adverse events [30]. However, treating AD with young plasma may involve multidose of large volumes of plasma infusion, which could have potential risks, including allergic reactions, infectious disease transmission, transfusion-related acute lung injury or transfusion-associated circulatory overload, and other unknown consequences, especially if the recipients are aged AD patients that are more vulnerable to these risks. Given these potential risks associated with plasma administration, administration of plasma in humans need large sample size for clinical trials to assess its feasibility and effectiveness.

## Conclusions

The present study shows that circulatory factors in young plasma can reduce neuroinflammation, reduce the deposition of Aβ, decrease the level of tau hyperphosphorylation, and reverse the cognitive impairment in aged 3×Tg-AD mice. These findings provide an initial evidence to support young plasma treatment for the rescue of cognitive impairment in AD and support further clinical trials in patients with AD and other neurodegenerative diseases.

## Supplementary information

**Additional file 1: Supplementary Figure 1.** Western blots of hippocampal homogenates from 3xTg-AD mice after treatment with young blood plasma or saline (Control). A, Western blots developed with antibodies against the synaptic marker proteins, CREB and NeuN as indicated on the left of the blots. **B,** Densitometric quantification of the blots after normalization with GAPDH. Unpaired t test was used for analysis. *N* = 10 for young plasma treatment, *n* = 11 for vehicle control (saline).

## Data Availability

The datasets used and/or analyzed during the current study are available from the corresponding author on reasonable request.

## Abbreviations

AD: Alzheimer’s disease
Aβ: Amyloid-β
NFTs: Neurofibrillary tangles
3×Tg-AD: Triple-transgenic Alzheimer’s disease mouse model
CREB: cAMP response element-binding protein
CNS: Central nervous system
CCL11: C-C motif chemokine 11
TIMP2: Tissue inhibitor of metalloproteinases 2
GHRH: Growth hormone–releasing hormone
CNTF: Ciliary neurotrophic factor
GDF11: Growth differentiation factor 11
