# Supplementary material for: Young blood plasma reduces Alzheimer’s disease-like brain pathologies and ameliorates cognitive impairment in 3×Tg-AD mice
Source: Alzheimers Res Ther. 2020 Jun 8;12:70. doi: 10.1186/s13195-020-00639-w (PMC7278124; doi:10.1186/s13195-020-00639-w)
Supplement: Supplementary file 1 — Additional file 1: Supplementary Figure 1. Western blots of hippocampal homogenates from 3xTg-AD mice after treatment with young blood plasma or saline (Control). A, Western blots developed with antibodies against the synaptic marker proteins, CREB and NeuN as indicated on the left of the blots. B, Densitometric quantification of the blots after normalization with GAPDH. Unpaired t test was used for analysis. N = 10 for young plasma treatment, n = 11 for vehicle control (saline). [file 13195_2020_639_MOESM1_ESM.docx]

**Young blood plasma reduces Alzheimer’s disease-like brain pathologies and ameliorates cognitive impairment in 3xTg-AD mice**

Ying Zhao^1,2,#^, Ran Qian^1,3,#^, Jin Zhang^1,4^, Fei Liu^1^, Khalid Iqbal^1^, Chun-Ling Dai^1,*^, Cheng-Xin Gong^1,*^

**Supplementary Fig 1**. Western blots of hippocampal homogenates from 3xTg-AD mice after treatment with young blood plasma or saline (Control). A, Western blots developed with antibodies against the synaptic marker proteins, CREB and NeuN as indicated on the left of the blots. **B,** Densitometric quantification of the blots after normalization with GAPDH. Unpaired t test was used for analysis. N = 10 for young plasma treatment, n=11 for vehicle control (saline).
